# Data-driven blood glucose level prediction in type 1 diabetes: a comprehensive comparative analysis

**DOI:** 10.1038/s41598-024-70277-x

**Published:** 2024-09-19

**Authors:** Hoda Nemat, Heydar Khadem, Jackie Elliott, Mohammed Benaissa

**Affiliations:** 1 Department of Electronic and Electrical Engineering, University of Sheffield, Sheffield, S1 3JD UK; 2 Department of Oncology and Metabolism, University of Sheffield, Sheffield, S10 2RX UK; 3 Diabetes and Endocrine Centre, Northern General Hospital, Sheffield Teaching Hospitals, Sheffield, S5 7AU UK

**Keywords:** Biomedical engineering, Computer science

## Abstract

Accurate prediction of blood glucose level (BGL) has proven to be an effective way to help in type 1 diabetes management. The choice of input, along with the fundamental choice of model structure, is an existing challenge in BGL prediction. Investigating the performance of different data-driven time series forecasting approaches with different inputs for BGL prediction is beneficial in advancing BGL prediction performance. Limited work has been made in this regard, which has resulted in different conclusions. This paper performs a comprehensive investigation of different data-driven time series forecasting approaches using different inputs. To do so, BGL prediction is comparatively investigated from two perspectives; the model’s approach and the model’s input. First, we compare the performance of BGL prediction using different data-driven time series forecasting approaches, including classical time series forecasting, traditional machine learning, and deep neural networks. Secondly, for each prediction approach, univariate input, using BGL data only, is compared to a multivariate input, using data on carbohydrate intake, injected bolus insulin, and physical activity in addition to BGL data. The investigation is performed on two publicly available Ohio datasets. Regression-based and clinical-based metrics along with statistical analyses are performed for evaluation and comparison purposes. The outcomes show that the traditional machine learning model is the fastest model to train and has the best BGL prediction performance especially when using multivariate input. Also, results show that simply adding extra variables does not necessarily improve BGL prediction performance significantly, and data fusion approaches may be required to effectively leverage other variables’ information.

## Introduction

It is essential to maintain a normal blood glucose level (BGL) when managing type 1 diabetes mellitus (T1DM)^1^. To aid this, one application of artificial intelligence is to predict the BGL of individuals with T1DM utilising the current and past information^2^. An early warning system for insufficient glycaemic control can be provided by BGL prediction^3^. However, this prediction is challenging because of some of the physiological factors such as the delay in food and insulin absorption, considerable variation between and within patients, and the complexity of interference factors such as physical activity^4,5^. Hence, despite all the research performed in the field of BGL prediction, accurate predictions remain a challenge^6^.

Based on the model structure and knowledge requirements, there are three main types of BGL prediction algorithms: physiological models (extensive knowledge), hybrid models (intermediate knowledge), and data-driven models (black-box approaches)^2,7,8^. Data-driven models have attracted considerable attention and are being increasingly explored. These models can be classified into classical time series forecasting (CTF), traditional machine learning (TML), and deep neural network (DNN) approaches. Comparing the efficacy of various data-driven prediction models using different approaches would be beneficial in the advancement of BGL prediction performance. However, using different datasets, different inputs, and different model settings has made this comparison difficult and limited studies have been published in this regard. Xie and Wang^6^ benchmarked a classical autoregression with an exogenous input model against ten different machine learning models for BGL prediction in T1DM patients. Zhang et al.^9^, also, compared four different data-driven models to forecast BGL in T1DM. They found that while their sequence-to-sequence Long short-term memory (LSTM) model was the most accurate at BGL prediction 30 minutes, in advance, their multiple linear regression model performed best to predict BGL 60 minutes, in advance. Moreover, Rodriguez et al.^10^ compared four different prediction models (two TNL and two DNN). According to the $R^2$ and RMSE metrics, they introduced the Bayesian neural network as the best model for BGL prediction.

Another essential factor for categorising BGL prediction algorithms is the input^7,11^. Common inputs of BGL prediction models are the current and past information on BGL, carbohydrate (Carb), bolus insulin (Bolus), and physical activity^12^. There is some evidence that BGL prediction from CGM data alone facilitates practical application in the real world therefore suggesting that there is no need for the extra effort and cost to acquire and process data from several sensors and modalities. Hence, several studies^13–20^ tried to predict BGL using CGM data, only. Conversely, there is evidence that other variables can also contribute to the performance of BGL prediction. Hence, other studies used exogenous data, such as Carb, Bolus, and physical activity, along with BGL data^21–25^. Investigating to what extent other relevant variables can contribute to the performance of BGL prediction in different time series forecasting approaches would be another helpful factor in the advancement of BGL prediction. Limited attempts have been made to compare different inputs, which has resulted in different conclusions. Zecchin et al.^26^ showed that adding Carb and Bolus data to CGM data can Predict BGL more accurately using a neural network in a prediction horizon between 30 and 120 minutes. Also, Nordin et al.^27^, using an LSTM model, showed superior performance of the multivariate model compared to the univariate model. While Hameed et al.^28^ concluded that whilst adding more information about Carb and Bolus adds more perturbations, it does not always improve the accuracy of prediction.

Previous studies have not provided an in-depth and comprehensive comparison of different prediction approaches or inputs. In addition, the average prediction performance across the data providers was considered for the purpose of comparison. However, it would not be meaningful to compare the averages of different data sets if they are not comparable^29^. Since there is a considerable variation between patients regarding BGL^5^, for a more valid comparison, statistical analyses need also to be considered. Hence, due to the lack of statistical analyses in the previous studies, their conclusions may not be robust. This work comprehensively investigates the performance of different personalised data-driven time series forecasting approaches for BGL prediction using univariate input (BGL data only) and multivariate input (BGL data along with Carb, Bolus, and physical activity data) separately. Also, a comparison between univariate and multivariate inputs is performed for each prediction approach to investigate to what extent other relevant variables than BGL can contribute to the performance of BGL prediction in prediction horizons of 30 and 60 minutes using different time series forecasting approaches. In order to strengthen evidence in this area, regression-based and clinical-based metrics are used to evaluate the performance of different cases. Rigorous statistical analyses are then applied to compare and contrast different models’ performance and the effect of additional inputs. The analyses are performed using two approved, publicly available Ohio datasets^30,31^.

## Material and methods

This section gives a brief description of the datasets used, data preprocessing steps, and the developed prediction models from different time series forecasting approaches. Subsequently, applied evaluation and statistical analyses are presented.

### Dataset

According to the review performed by Felizardo et al.^32^, the Ohio T1DM dataset^30,31^ with replication capability is the most frequently used clinical dataset in the literature that is publicly accessible. Hence, to do a reliable comparison, in this study, we used the Ohio T1DM dataset. The Ohio T1DM dataset comprises two sets of data from 12 people with T1DM. The first dataset related to six T1DM patients was released in 2018 for the first BGL prediction challenge^33^ (called Ohio_2018). The second dataset related to an additional six patients was released in 2020 for the second BGL prediction challenge^34^ (called Ohio_2020). Data contributors comprised five females and seven males and were aged 20 to 80 years at data collection time. Table 1 provides the details related to the gender and age range of participants in both cohorts.

**Table 1:** Information about the gender and age of contributors to the Ohio_2018 and Ohio_2020 datasets.

Ohio_2018			Ohio_2020		
PID	Gender	Age	PID	Gender	Age
559	Female	40–60	540	Male	20–40
563	Male	40–60	544	Male	40–60
570	Male	40–60	552	Male	20–40
575	Female	40–60	567	Female	20–40
588	Female	40–60	584	Male	40–60
591	Female	40–60	596	Male	60–80

*PID* patient identity.

An insulin pump, a CGM sensor, and a fitness band were used by the patients. Along with physiological sensors, each individual reported Carb estimations, Bolus, and life events. Participants in both cohorts used a Medtronic Enlite CGM sensor for measuring their BGL. In the Ohio_2018 dataset, patients wore Basis Peak fitness bands that collected heart rate (HR) data, and patients in the Ohio_2020 cohort wore Empatica Embrace fitness bands collecting magnitude of acceleration (MA) data. Data were collected over an eight-week period by allocating the last 10 days for testing sets and the rest for training sets. BGL data from CGM sensors and HR data from the Basis Peak band were collected with a 5-minute aggregation. Data of MA from the Empatica Embrace band was collected every minute. Further information about the data collection can be found in^30,31^. In this study, automatically collected BGL and activity data and self-reported Carb and Bolus data are used.

### Preprocessing

**Figure 1:**
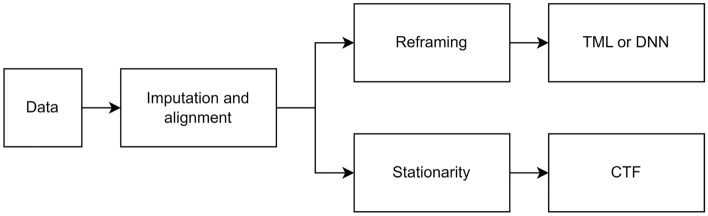
A schematic diagram demonstrating the preprocessing steps.

There were some mandatory preprocessing steps to overcome many imperfections and missing data when analysing real-world data. Additionally, some data preprocessing was required depending on the forecasting approach used (Fig. 1).

#### Imputation and alignment

The initial preprocessing step was to address the issue of missing BGL and physical activity data. These missing values were interpolated in training and extrapolated in testing sets linearly. No reported timestamps for Carb and Bolus data were assigned to zero. The following preprocessing step was to align the BGL data with other data. Data of MA, with a resolution of one minute, was downsampled to a resolution of five minutes by taking the nearest MA data point with a BGL data point and removing the remainder. The HR data, which had the same resolution as BGL data, only required to be aligned. Additionally, the unavailable data timestamps at the beginning/ending of each set, which occurred due to different times in the wearing sensors, were discarded.

#### Stationarity

When applying the CTF approach, two common statistical tests were applied to check the primary assumption of stationarity^35^; the Augmented Dickey-Fuller (ADF) test^36^ and the Kwiatkowski-Phillips-Schmidt-Shin (KPSS) test^37^. Time series in which both tests confirmed the stationarity were defined as stationary. Since the ADF test indicated stationarity for all variables and all patients, integrated differencing was applied to the time series in which the KPSS indicated non-stationarity.

#### Reframing

When applying TML or DNN approaches, the multi-ahead time series forecasting problem should be reframed as a supervised learning task. To accomplish this, historical observations were used as inputs, and future observations were used as outputs.

### Time series forecasting approaches

To comprehensively investigate and compare the performance of BGL prediction, different time series forecasting categories, including CTF, TML, and DNN, were examined. Also, following the BGL prediction challenges in which the participants were asked to predict BGL 30 and 60 min in advance and in line with many papers in the literature^6,18,25^, 30 and 60 min prediction horizons were considered. There is a pool of models for BGL prediction in each category. For the sake of feasibility and in order to minimise the complexity of comparison, for each category, a common successful model found in the literature was developed and fine-tuned as a representative. For input comparison purposes, each model was first trained as a univariate prediction model; then, its counterpart was developed as a multivariate prediction model. The prediction models are briefly described in the following.

#### Classical time series forecasting (CTF)

CTF is a common approach for the BGL prediction task^6,38^. One of the most commonly used models in this category is the autoregressive integrated moving average (ARIMA)^39^. ARIMA is a combination of linear processes of autoregression (AR) and moving average (MA) models, as well as integrated differencing. It models the future as a linear combination of lags and lagged residual errors in a differenced time series in the case of non-stationarity. To develop an ARIMA model, the parameters of the model, including p (AR order), d (differencing order), and q (MA order), should be determined. The p and q parameters were optimised for each patient automatically by examining each parameter from zero to 36. The d parameter was also determined by considering the stationarity tests. An autoregressive integrated moving average with exogenous variables (ARIMAX) was used for the multivariate prediction, incorporating exogenous variables into the univariate ARIMA model. Table 2 shows the optimised parameters for each patient training the ARIMA and ARIMAX models.

**Table 2:** The optimised parameters for the ARIMA and ARIMAX models.

Ohio_2018				Ohio_2020			
PID	p	d	q	PID	p	d	q
559	6	0	2	540	4	1	2
563	3	0	2	544	5	1	3
570	3	0	2	552	3	1	1
575	4	1	3	567	1	1	2
588	1	1	1	584	2	0	3
591	2	0	4	596	3	1	1

*PID* patient identity.

#### Traditional machine learning (TML)

A TML approach has also received significant attention for predicting BGL. Support vector machines (SVMs) have been shown to be the most accurate in the BGL prediction task among different classes of machine learning algorithms^5,40^. Also, among different types of SVMs, support vector regression (SVR) is the most commonly employed technique for predicting BGL^5^. In this study, in line with the successfully developed SVM model for BGL prediction in the literature^41^, an SVR model with a radial basis kernel was developed. Moreover, vectorised multivariate data were utilised as the input for developing multivariate counterparts to have a multivariate prediction using SVM. The hyperparameters of the SVR model, including gamma, C, and epsilon, were chosen using a grid search during a tuning process for each patient and each input. Search spaces of {0.1,1, 10, 100}, {0.001,0.01, 0.1, 1}, and {0.01, 0.1, 1, 10} were explored to optimise gamma, C, and epsilon parameters, respectively. The chosen parameters are summarised in Table 3.

**Table 3:** The optimised parameters for the SVR model.

	PID	Univariate						Multivariate					
3-14		PH:30 min			PH:60 min			PH:30 min			PH:60 min		
3-14		$\gamma$	c	$\epsilon$	$\gamma$	c	$\epsilon$	$\gamma$	c	$\epsilon$	$\gamma$	c	$\epsilon$
Ohio_2018	559	100	10	1	100	10	1	100	10	0.1	100	10	0.1
	563	100	10	0.1	100	10	0.1	100	0.01	0.1	100	10	0.1
	570	100	1	1	10	1	1	100	1	0.1	100	10	0.1
	575	100	0.01	1	100	10	1	100	0.01	0.1	100	10	0.1
	588	100	10	0.1	100	10	1	100	1	0.1	100	10	0.1
	591	100	10	1	10	0.01	1	100	10	0.1	100	10	0.1
Ohio_2020	540	100	10	1	100	10	1	100	10	0.1	100	10	0.1
	544	100	10	1	100	10	1	100	1	0.01	100	10	0.1
	552	100	10	1	100	10	1	100	10	0.1	100	10	0.1
	567	100	10	1	100	10	1	100	10	0.1	100	10	1
	584	100	10	1	100	10	1	100	10	0.1	100	10	0.1
	596	100	10	1	100	1	0.1	100	10	0.1	100	10	1

*PID* patient identity, *PH* prediction horizon.

#### Deep neural network (DNN)

As a class of recurrent neural networks, LSTM networks are effective at predicting BGL based on sequential data^42–45^. In this study, the sequence-to-sequence forecasting task was carried out using an LSTM model recently developed by our team, which has been optimised in the Ohio datasets^13,21^. The vanilla LSTM network consisted of an LSTM layer, a dense layer, and an output layer. The initialiser of He uniform, the activation function of ReLU, the optimiser of Adam, and the loss function of mean square error were chosen. Also, an epoch size of 200 and a batch size of 32 were selected. An initial learning rate of 0.01 was reduced by 0.1 following the usage of a ReduceLROnPlateau callback with patience of 20 after stopping validation loss improvement.

### Evaluation criteria

In this work, two regression-based and clinically-based evaluation criteria were examined to comprehensively investigate BGL prediction performance based on different prediction approaches and inputs. The following subsections provide a brief description of these criteria.

#### Regression-based criteria

According to Eqs. (1) and (2), the overall performance of BGL prediction models was evaluated based on root mean square error (RMSE) and mean absolute error (MAE), as two commonly used regression accuracy metrics in BG-related works^46–49^.

1 $$\begin{aligned} RMSE= &   \sqrt{\frac{\sum _{i=1}^{N} (y_i-\hat{y_i})^2}{N}} \end{aligned}$$

2 $$\begin{aligned} MAE= &   \frac{\sum _{i=1}^{N} |y_i-\hat{y_i}|}{N} \end{aligned}$$

In both equations, *N* represents the evaluation set size, $y_i$ represents the reference, and $\hat{y_i}$ represents the prediction.

#### Clinical-based criteria

The clinical performance of each model was evaluated using the Matthews correlation coefficient (MCC) and surveillance error (SE), which have recently been used for clinical evaluation of BGL prediction^18,43,44^. The MCC criterion was used to measure whether the models could accurately distinguish adverse glycaemic events from normoglycaemic events. Using SE metric, an average of the surveillance error grid^50^ interpolated bilinearly, each patient was assigned a unique score.

### Statistical analyses

The BGL prediction performance measured by evaluation metrics with various prediction approaches or inputs was also statistically analysed over data contributors for each dataset. In accordance with the conditions of each comparison, appropriate statistical analyses were conducted.

To compare different prediction models, firstly, the Friedman test^51^ was conducted in order to find out whether at least two approaches differ significantly (with a significance level of five percent). If this was the case, the post-hoc Nemenyi test^52^ was then performed comparing different approaches’ performance in a pair-wise fashion. Also, since multiple comparisons were made, the Holm procedure^53^ was applied to correct the significance level. A critical difference (CD) diagram^29^ was drawn to illustrate the results of each post-hoc test. These analyses were performed for each univariate and multivariate input separately.

To compare univariate and multivariate inputs for each prediction approach, the non-parametric Wilcoxon signed-ranks test^54^, which is an appropriate test for comparing two approaches without the assumption of normality, was applied^29^. This test, with a significance level of five percent was conducted to check the consistency of each evaluation metric calculated for univariate and multivariate inputs over the data contributors of each dataset. The comparison of input was performed for each prediction approach separately.

## Results and discussion

In this section, firstly the evaluation results for both Ohio_2018 and Ohio_2020 datasets and 30-minute and 60-minute prediction horizons are presented. Then, depending on which factor is being compared, the results of relative statistical analyses are presented and discussed in two parts; comparing models’ approaches and models’ inputs.

### Evaluation results

Tables 4 and 5 provide the evaluation results for BGL prediction models related to different approaches for both univariate and multivariate inputs, 30 and 60 min in advance in Ohio_2018 dataset, respectively. Also, Tables 6 and 7 provide the evaluation results in the Ohio_2020 dataset, for prediction horizons of 30 and 60 min, respectively. It is worth noting that for the DNN approach, due to the random initialization, the average and standard deviation of evaluation results over 10 runs are reported. Using evaluation results, to compare different models and inputs, statistical analyses were performed. The results are discussed in the following sections.

**Table 4:** Evaluation results of different prediction approaches and inputs in Ohio_2018 dataset for prediction horizons of 30 min.

PID	Model	Input	RMSE	MAE	MCC	SE
559	CTF	Univariate	20.07	13.82	0.78	0.19
		Multivariate	20.12	13.86	0.79	0.20
	TML	Univariate	20.56	14.00	0.81	0.19
		Multivariate	19.35	13.34	0.83	0.18
	DNN	Univariate	20.19 ± 0.18	14.16 ± 0.13	0.78 ± 0.01	0.21 ± 0.01
		Multivariate	20.70 ± 0.41	14.68 ± 0.31	0.80 ± 0.01	0.20 ± 0.01
563	CTF	Univariate	20.14	13.82	0.75	0.20
		Multivariate	20.33	13.98	0.75	0.20
	TML	Univariate	18.67	13.28	0.75	0.19
		Multivariate	18.52	12.89	0.77	0.18
	DNN	Univariate	18.93 ± 0.10	13.12 ± 0.13	0.77 ± 0.01	0.18 ± 0.00
		Multivariate	20.45 ± 0.32	14.11 ± 0.24	0.76 ± 0.01	0.19 ± 0.00
570	CTF	Univariate	17.01	12.17	0.86	0.12
		Multivariate	17.15	12.32	0.85	0.12
	TML	Univariate	17.24	11.71	0.87	0.11
		Multivariate	16.09	11.20	0.87	0.10
	DNN	Univariate	17.11 ± 0.52	11.97 ± 0.45	0.87 ± 0.01	0.11 ± 0.00
		Multivariate	18.10 ± 0.40	12.58 ± 0.24	0.86 ± 0.01	0.12 ± 0.00
575	CTF	Univariate	25.17	15.58	0.76	0.23
		Multivariate	25.17	15.58	0.76	0.23
	TML	Univariate	24.08	14.93	0.74	0.22
		Multivariate	24.08	14.93	0.76	0.22
	DNN	Univariate	24.42 ± 0.21	15.72 ± 0.24	0.73 ± 0.01	0.24 ± 0.01
		Multivariate	25.79 ± 0.49	15.78 ± 0.39	0.72 ± 0.01	0.23 ± 0.01
588	CTF	Univariate	19.62	14.19	0.74	0.19
		Multivariate	19.62	14.20	0.74	0.19
	TML	Univariate	21.28	15.34	0.69	0.20
		Multivariate	18.03	13.09	0.75	0.17
	DNN	Univariate	18.84 ± 0.10	13.54 ± 0.07	0.75 ± 0.01	0.18 ± 0.00
		Multivariate	18.84 ± 0.35	13.80 ± 0.34	0.76 ± 0.01	0.18 ± 0.00
591	CTF	Univariate	22.65	16.03	0.66	0.27
		Multivariate	22.69	16.06	0.65	0.27
	TML	Univariate	21.78	15.61	0.65	0.27
		Multivariate	21.49	15.50	0.65	0.26
	DNN	Univariate	22.87 ± 0.45	16.59 ± 0.48	0.63 ± 0.01	0.29 ± 0.01
		Multivariate	22.79 ± 0.31	16.47 ± 0.27	0.64 ± 0.01	0.28 ± 0.01
Avg	CTF	Univariate	20.78	14.27	0.76	0.20
		Multivariate	20.85	14.33	0.76	0.20
	TML	Univariate	20.60	14.14	0.75	0.20
		Multivariate	19.59	13.49	0.77	0.19
	DNN	Univariate	20.39 ± 0.26	14.18 ± 0.25	0.75 ± 0.01	0.20 ± 0.00
		Multivariate	21.11 ± 0.38	14.57 ± 0.30	0.76 ± 0.01	0.20 ± 0.00

*PID* patient identity, *PH* prediction horizon, *RMSE* root mean square error, *MAE* mean absolute error, *MCC* Matthews correlation coefficient, *SE* surveillance error, *CTF* classical time series forecasting, *TML* traditional machine learning, *DNN* deep neural network.

**Table 5:** Evaluation results of different prediction approaches and inputs in Ohio_2018 dataset for prediction horizons of 60 min.

PID	Model	Input	RMSE	MAE	MCC	SE
559	CTF	Univariate	36.03	25.76	0.58	0.36
		Multivariate	36.24	26.00	0.58	0.36
	TML	Univariate	35.69	25.44	0.63	0.33
		Multivariate	31.69	22.51	0.69	0.29
	DNN	Univariate	35.83 ± 0.45	26.31 ± 0.27	0.62 ± 0.01	0.35 ± 0.01
		Multivariate	35.52 ± 0.80	26.02 ± 0.74	0.61 ± 0.02	0.35 ± 0.01
563	CTF	Univariate	33.01	24.39	0.54	0.34
		Multivariate	32.84	24.36	0.53	0.34
	TML	Univariate	30.32	22.13	0.54	0.31
		Multivariate	30.32	21.72	0.59	0.29
	DNN	Univariate	32.25 ± 1.22	23.45 ± 1.33	0.52 ± 0.05	0.32 ± 0.02
		Multivariate	33.63 ± 0.67	23.97 ± 0.54	0.54 ± 0.02	0.32 ± 0.01
570	CTF	Univariate	30.20	22.84	0.75	0.22
		Multivariate	30.37	23.01	0.74	0.22
	TML	Univariate	29.50	21.17	0.79	0.19
		Multivariate	27.67	19.98	0.79	0.18
	DNN	Univariate	29.02 ± 0.62	20.75 ± 0.62	0.80 ± 0.00	0.19 ± 0.00
		Multivariate	30.95 ± 0.46	22.23 ± 0.59	0.80 ± 0.01	0.20 ± 0.00
575	CTF	Univariate	39.96	27.51	0.56	0.41
		Multivariate	39.97	27.51	0.56	0.41
	TML	Univariate	37.09	25.98	0.51	0.39
		Multivariate	36.01	25.24	0.56	0.37
	DNN	Univariate	38.09 ± 0.30	27.10 ± 0.18	0.50 ± 0.01	0.41 ± 0.00
		Multivariate	40.02 ± 0.69	27.60 ± 0.29	0.51 ± 0.01	0.41 ± 0.00
588	CTF	Univariate	33.98	25.15	0.57	0.33
		Multivariate	33.98	25.16	0.57	0.33
	TML	Univariate	31.43	22.73	0.56	0.29
		Multivariate	30.21	22.28	0.59	0.28
	DNN	Univariate	31.62 ± 0.16	23.24 ± 0.15	0.54 ± 0.01	0.31 ± 0.00
		Multivariate	31.91 ± 0.42	23.31 ± 0.34	0.58 ± 0.02	0.30 ± 0.00
591	CTF	Univariate	36.94	27.53	0.36	0.46
		Multivariate	36.98	27.57	0.35	0.46
	TML	Univariate	33.58	25.40	0.45	0.41
		Multivariate	33.33	25.42	0.41	0.41
	DNN	Univariate	36.71 ± 0.80	28.77 ± 0.78	0.38 ± 0.02	0.46 ± 0.01
		Multivariate	35.69 ± 0.79	27.53 ± 0.67	0.44 ± 0.02	0.44 ± 0.01
Avg	CTF	Univariate	35.02	25.53	0.56	0.35
		Multivariate	35.06	25.60	0.56	0.36
	TML	Univariate	32.93	23.81	0.58	0.32
		Multivariate	31.54	22.86	0.61	0.30
	DNN	Univariate	33.92 ± 0.59	24.94 ± 0.55	0.56 ± 0.02	0.34 ± 0.01
		Multivariate	34.62 ± 0.64	25.11 ± 0.53	0.58 ± 0.01	0.34 ± 0.01

*PID* patient identity, *PH* prediction horizon, *RMSE* root mean square error, *MAE* mean absolute error, *MCC* Matthews correlation coefficient, *SE* surveillance error, *CTF* classical time series forecasting, *TML* traditional machine learning, *DNN* deep neural network.

**Table 6:** Evaluation results of different prediction approaches and inputs in Ohio_2020 dataset for prediction horizons of 30 min.

PID	Model	Input	RMSE	MAE	MCC	SE
540	CTF	Univariate	21.46	16.13	0.73	0.25
		Multivariate	22.01	16.24	0.74	0.25
	TML	Univariate	29.07	18.34	0.71	0.26
		Multivariate	23.11	16.83	0.71	0.26
	DNN	Univariate	22.58 ± 0.77	16.82 ± 0.45	0.71 ± 0.01	0.25 ± 0.01
		Multivariate	21.99 ± 0.89	16.33 ± 0.33	0.70 ± 0.01	0.26 ± 0.00
544	CTF	Univariate	18.93	13.42	0.77	0.19
		Multivariate	18.94	13.42	0.77	0.19
	TML	Univariate	18.11	12.98	0.79	0.19
		Multivariate	18.74	13.32	0.78	0.19
	DNN	Univariate	18.14 ± 0.12	12.90 ± 0.13	0.79 ± 0.00	0.19 ± 0.00
		Multivariate	19.04 ± 0.19	13.07 ± 0.13	0.78 ± 0.01	0.19 ± 0.00
552	CTF	Univariate	17.42	12.30	0.74	0.21
		Multivariate	17.42	12.30	0.74	0.21
	TML	Univariate	17.01	12.47	0.74	0.21
		Multivariate	16.88	12.88	0.70	0.23
	DNN	Univariate	16.89 ± 0.05	12.49 ± 0.10	0.74 ± 0.01	0.21 ± 0.00
		Multivariate	18.48 ± 0.77	13.55 ± 0.54	0.70 ± 0.02	0.23 ± 0.01
567	CTF	Univariate	22.39	15.53	0.71	0.24
		Multivariate	22.39	15.53	0.71	0.24
	TML	Univariate	21.06	14.84	0.67	0.25
		Multivariate	21.82	15.38	0.62	0.26
	DNN	Univariate	21.22 ± 0.21	15.11 ± 0.23	0.65 ± 0.01	0.26 ± 0.00
		Multivariate	20.87 ± 0.30	14.67 ± 0.23	0.65 ± 0.02	0.25 ± 0.00
584	CTF	Univariate	22.53	16.06	0.74	0.22
		Multivariate	23.36	16.81	0.73	0.23
	TML	Univariate	21.88	15.84	0.77	0.22
		Multivariate	21.23	15.40	0.78	0.21
	DNN	Univariate	23.16 ± 0.50	17.02 ± 0.43	0.76 ± 0.01	0.23 ± 0.00
		Multivariate	22.66 ± 0.59	16.56 ± 0.46	0.77 ± 0.01	0.23 ± 0.01
596	CTF	Univariate	18.88	13.50	0.71	0.22
		Multivariate	18.88	13.50	0.71	0.22
	TML	Univariate	17.89	12.76	0.74	0.21
		Multivariate	16.86	12.21	0.78	0.19
	DNN	Univariate	18.17 ± 0.11	12.94 ± 0.10	0.75 ± 0.01	0.21 ± 0.00
		Multivariate	18.52 ± 0.38	13.11 ± 0.26	0.75 ± 0.01	0.21 ± 0.00
Avg	CTF	Univariate	20.27	14.49	0.73	0.22
		Multivariate	20.50	14.63	0.73	0.23
	TML	Univariate	20.83	14.54	0.74	0.22
		Multivariate	19.77	14.34	0.73	0.22
	DNN	Univariate	20.03 ± 0.30	14.55 ± 0.24	0.73 ± 0.01	0.23 ± 0.00
		Multivariate	20.26 ± 0.52	14.55 ± 0.33	0.72 ± 0.01	0.23 ± 0.00

*PID* patient identity, *PH* prediction horizon, *RMSE* root mean square error, *MAE* mean absolute error, *MCC* Matthews correlation coefficient, *SE* surveillance error, *CTF* classical time series forecasting, *TML* traditional machine learning, *DNN* deep neural network.

**Table 7:** Evaluation results of different prediction approaches and inputs in Ohio_2020 dataset for prediction horizons of 60 min.

PID	Model	Input	RMSE	MAE	MCC	SE
540	CTF	Univariate	40.42	31.13	0.52	0.46
		Multivariate	42.54	32.46	0.51	0.48
	TML	Univariate	44.81	32.49	0.50	0.45
		Multivariate	41.42	30.90	0.54	0.44
	DNN	Univariate	40.83 ± 1.33	30.98 ± 0.45	0.53 ± 0.01	0.44 ± 0.00
		Multivariate	41.75 ± 0.88	31.00 ± 0.48	0.53 ± 0.03	0.44 ± 0.01
544	CTF	Univariate	34.84	25.36	0.57	0.36
		Multivariate	34.85	25.35	0.57	0.36
	TML	Univariate	32.01	23.42	0.61	0.33
		Multivariate	28.25	20.49	0.66	0.30
	DNN	Univariate	32.00 ± 0.21	24.69 ± 0.32	0.60 ± 0.01	0.36 ± 0.01
		Multivariate	32.33 ± 1.07	22.74 ± 0.69	0.64 ± 0.02	0.33 ± 0.01
552	CTF	Univariate	32.13	22.61	0.57	0.37
		Multivariate	32.13	22.61	0.57	0.37
	TML	Univariate	29.76	21.49	0.58	0.34
		Multivariate	28.87	21.87	0.58	0.35
	DNN	Univariate	30.32 ± 0.13	22.71 ± 0.17	0.58 ± 0.01	0.36 ± 0.00
		Multivariate	30.98 ± 0.65	23.47 ± 0.54	0.56 ± 0.02	0.37 ± 0.01
567	CTF	univariate	42.34	30.13	0.48	0.46
		Multivariate	42.34	30.13	0.48	0.46
	TML	Univariate	37.16	27.31	0.40	0.44
		Multivariate	37.46	27.40	0.44	0.44
	DNN	Univariate	39.23 ± 1.86	30.28 ± 2.12	0.36 ± 0.02	0.51 ± 0.04
		Multivariate	36.63 ± 0.13	27.42 ± 0.22	0.38 ± 0.01	0.47 ± 0.00
584	CTF	Univariate	38.93	28.07	0.56	0.37
		Multivariate	39.92	28.84	0.56	0.38
	TML	Univariate	36.77	27.11	0.63	0.35
		Multivariate	33.89	25.28	0.63	0.34
	DNN	Univariate	39.83 ± 1.96	30.16 ± 1.78	0.59 ± 0.03	0.40 ± 0.02
		Multivariate	38.38 ± 1.71	29.40 ± 1.76	0.57 ± 0.03	0.40 ± 0.02
596	CTF	Univariate	33.20	24.29	0.51	0.38
		multivariate	33.20	24.28	0.51	0.38
	TML	univariate	30.27	22.18	0.57	0.33
		Multivariate	27.82	20.15	0.61	0.30
	DNN	Univariate	30.20 ± 0.21	22.22 ± 0.25	0.58 ± 0.02	0.33 ± 0.01
		Multivariate	30.38 ± 1.07	22.45 ± 0.98	0.57 ± 0.03	0.33 ± 0.01
Avg	CTF	Univariate	36.98	26.93	0.53	0.40
		Multivariate	37.50	27.28	0.53	0.40
	TML	Univariate	35.13	25.67	0.55	0.38
		Multivariate	32.95	24.35	0.58	0.36
	DNN	Univariate	35.40 ± 0.95	26.84 ± 0.85	0.54 ± 0.01	0.40 ± 0.01
		Multivariate	35.07 ± 0.92	26.08 ± 0.78	0.54 ± 0.02	0.39 ± 0.01

*PID* patient identity, *PH* prediction horizon, *RMSE* root mean square error, *MAE* mean absolute error, *MCC* Matthews correlation coefficient, *SE* surveillance error, *CTF* classical time series forecasting, *TML* traditional machine learning, *DNN* deep neural network.

**Figure 2:**
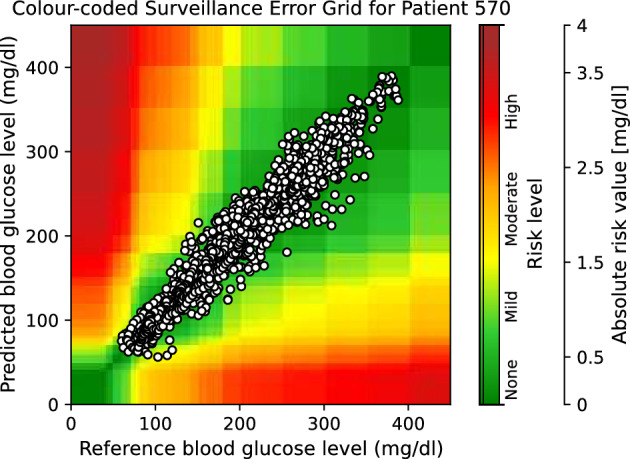
The colour-coded surveillance error grid related to the predictions of CTF approach with univariate input 30 min in advance for patient 570.

**Figure 3:**
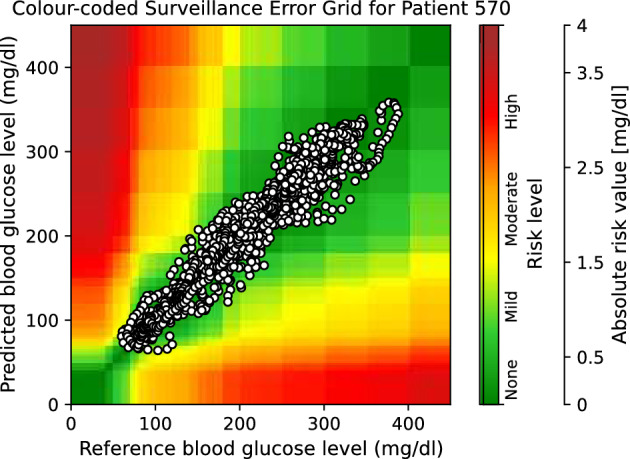
The colour-coded surveillance error grid related to the predictions of TML approach with univariate input 30 min in advance for patient 570.

**Figure 4:**
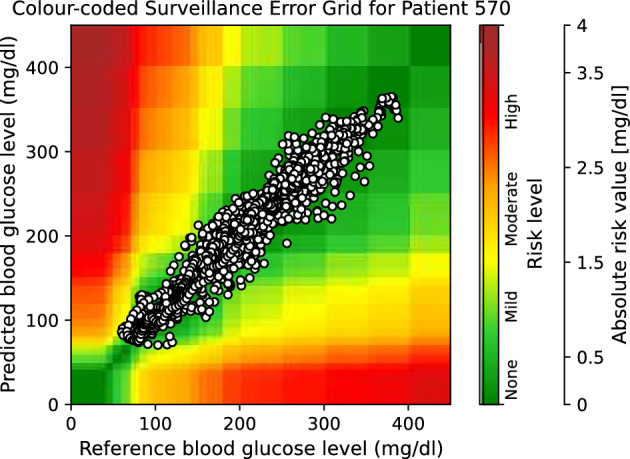
The colour-coded surveillance error grid related to the predictions of DNN approach with univariate input 30 min in advance for patient 570.

**Figure 5:**
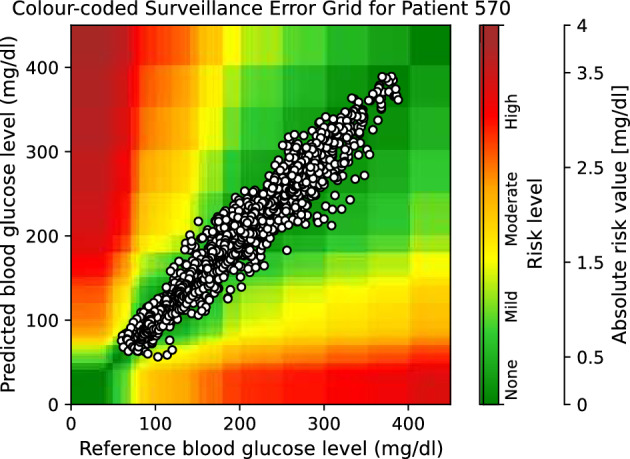
The colour-coded surveillance error grid related to the predictions of CTF approach with multivariate input 30 min in advance for patient 570.

**Figure 6:**
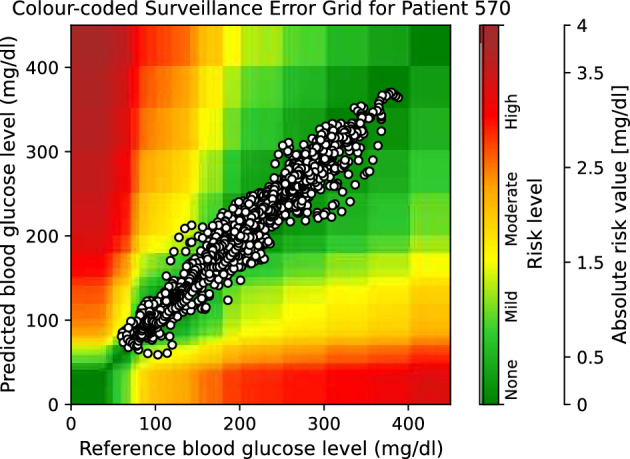
The colour-coded surveillance error grid related to the predictions of TML approach with multivariate input 30 min in advance for patient 570.

**Figure 7:**
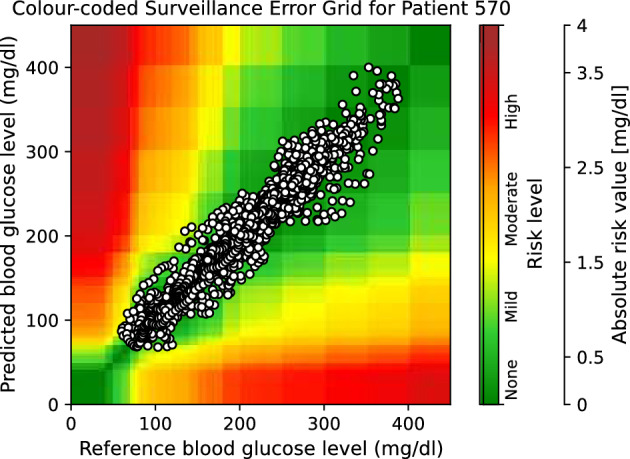
The colour-coded surveillance error grid related to the predictions of DNN approach with multivariate input 30 min in advance for patient 570.

Moreover, to provide visual clinical insight, colour-coded surveillance error grids are illustrated in Figs. 2, 3, 4, 5, 6 and 7, which are related to different models and inputs for BGL prediction 30 in advance for patient 570.

### Comparing models’ approaches

Different data-driven time series forecasting approaches are compared using univariate and multivariate inputs, separately. Firstly, the results of statistical analyses are presented and discussed. Secondly, computational costs for different models are compared. Then, according to all presented results, a conclusion is presented.

#### Statistical result

***Univariate input***

Table 8 presents p-values of the Friedman test calculated based on evaluation criteria using different BGL prediction approaches with a univariate input. The analysis was performed for both prediction horizons of 30 and 60 minutes, and for both Ohio_2018 and Ohio_2020 datasets, separately. With a significance level of five percent, p-values in bold font are related to the cases with probably at least one significant difference between the performance of models.

**Table 8:** p-values of the Friedman test for comparing all prediction models for univariate BGL prediction 30 and 60 minutes in advance in Ohio_2018 and Ohio_2020 datasets.

	PH	RMSE	MAE	MCC	SE
Ohio_2018	30 min	1.000	0.607	1.000	0.311
	60 min	**0.006**	**0.030**	0.513	**0.016**
Ohio_2020	30 min	0.223	0.607	0.311	0.607
	60 min	0.311	0.135	0.311	0.069

*PH* prediction horizon, *RMSE* root mean square error, *MAE* mean absolute error, *MCC* Matthews correlation coefficient, *SE* surveillance error.

**Figure 8:**
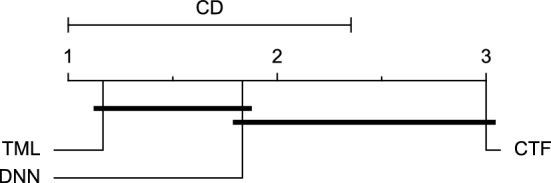
CD diagram of comparing different prediction models with univariate input pairwisely over the data contributors of Ohio_2018 dataset for the 60-minute prediction horizon based on RMSE metric.

**Figure 9:**
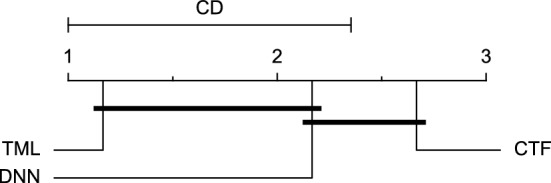
CD diagram of comparing different prediction models with univariate input pairwisely over the data contributors of Ohio_2018 dataset for the 60-min prediction horizon based on MAE metric.

**Figure 10:**
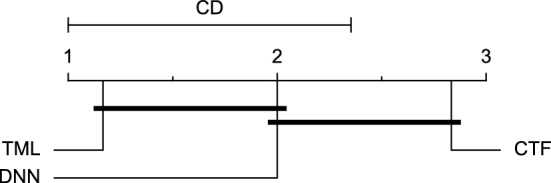
CD diagram of comparing different prediction models with univariate input pairwisely over the data contributors of Ohio_2018 dataset for the 60-minute prediction horizon based on SE metric.

Reviewing Tables 4, 5, 6, 7, and 8, it can be concluded that although there are differences between average evaluation metrics related to the performance of different prediction models over data providers of each cohort, these differences are mainly statistically insignificant. Table 8 shows that just three metrics of RMSE, MAE, and SE calculated for the 60-minute prediction horizon in the Ohio_2018 cohort may be significantly different between at least two prediction models. In those cases, the post-hoc Nemenyi test was performed for pair-wise comparisons between prediction models. Results of the Nemenyi tests are then visualised using CD diagrams, as shown in Figs. 8, 9, and 10, according to metrics RMSE, MAE, and SE, respectively. In each CD diagram, at a significance level of five percent, prediction models that differ insignificantly are linked by a horizontal line. It can be seen that while the TML model outperformed the CTF model significantly based on their average ranks for the examined metrics, the other pair-wise comparisons were not statistically meaningful.

#### Multivariate input

Using different forecasting approaches with multivariate input, Table 9 shows Friedman test p-values for each evaluation metric. The test was performed separately for each prediction horizon of 30 and 60 min and in each cohort. The p-values marked in bold font are considered significant at a significance level of five percent, showing that at least two prediction models may differ in the BGL prediction performance.

**Table 9:** p-values of the Friedman test for comparing all prediction models for multivariate BGL prediction 30 and 60 min in advance in Ohio_2018 and Ohio_2020 datasets.

	PH	RMSE	MAE	MCC	SE
Ohio_2018	30 min	**0.006**	**0.006**	0.115	**0.011**
	60 min	**0.011**	**0.009**	**0.030**	**0.006**
Ohio_2020	30 min	0.223	0.607	0.846	1.000
	60 min	**0.006**	**0.009**	**0.042**	**0.011**

*PH* prediction horizon, *RMSE* root mean square error, *MAE* mean absolute error, *MCC* Matthews correlation coefficient, *SE* surveillance error.

Considering the presented results in Table 9 and a significance level of five percent, it can be inferred that among different examined cases for comparing prediction approaches regarding evaluation metrics, prediction horizons, and datasets, at least two prediction approaches may perform differently for BGL prediction 60 minutes in advance in both Ohio_2018 and Ohio_2020 datasets based on all the evaluation metrics. Also, there are significant p-values for comparing different prediction models for the 30-minute prediction horizon in the Ohio_2018 dataset based on RMSE, MAE, and SE metrics.

The post-hoc Nemenyi test was conducted for each mentioned case to compare the prediction models in a pair-wise manner. The results of post-hoc tests are graphically presented in CD diagrams, as demonstrated in Figs. 11, 12, 13, 14, 15, 16, 17, 18, 19, 20 and 21. A horizontal line connects prediction models that differ insignificantly (with a significance level of five percent).

**Figure 11:**
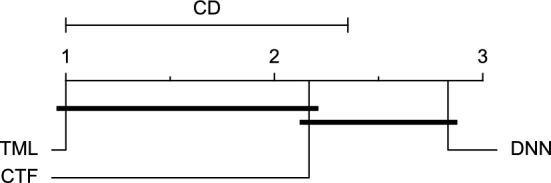
CD diagram of comparing different prediction models with multivariate input pairwisely over the data contributors of Ohio_2018 dataset for the 30-min prediction horizon based on RMSE metric.

**Figure 12:**
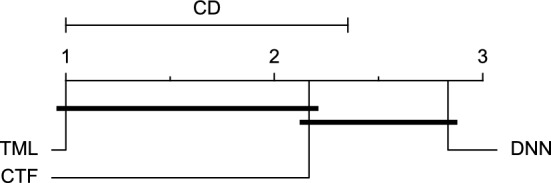
CD diagram of comparing different prediction models with multivariate input pairwisely over the data contributors of Ohio_2018 dataset for the 60-min prediction horizon based on MAE metric.

**Figure 13:**
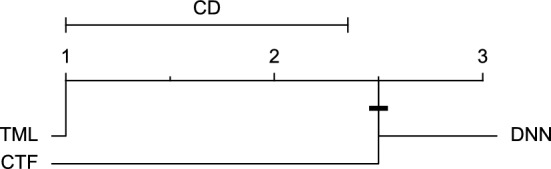
CD diagram of comparing different prediction models with multivariate input pairwisely over the data contributors of Ohio_2018 dataset for the 30-min prediction horizon based on SE metric.

**Figure 14:**
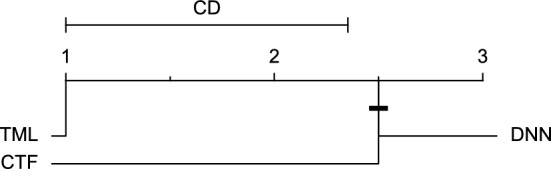
CD diagram of comparing different prediction models with multivariate input pairwisely over the data contributors of Ohio_2018 dataset for the 60-min prediction horizon based on RMSE metric.

**Figure 15:**
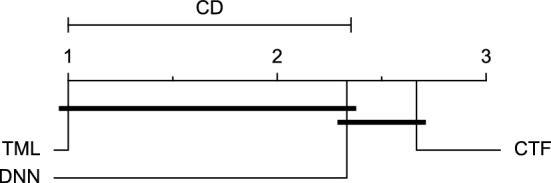
CD diagram of comparing different prediction models with multivariate input pairwisely over the data contributors of Ohio_2018 dataset for the 60-min prediction horizon based on MAE metric.

**Figure 16:**
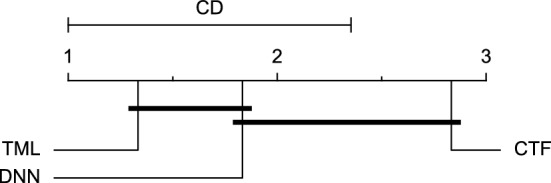
CD diagram of comparing different prediction models with multivariate input pairwisely over the data contributors of Ohio_2018 dataset for the 60-min prediction horizon based on MCC metric.

**Figure 17:**
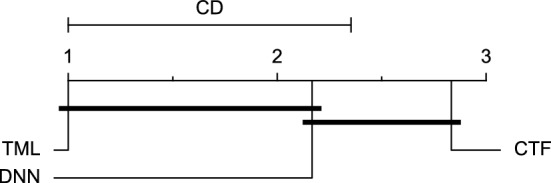
CD diagram of comparing different prediction models with multivariate input pairwisely over the data contributors of Ohio_2018 dataset for the 60-min prediction horizon based on SE metric.

**Figure 18:**
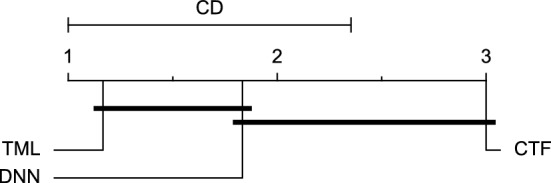
CD diagram of comparing different prediction models with multivariate input pairwisely over the data contributors of Ohio_2020 dataset for the 60-min prediction horizon based on RMSE metric.

**Figure 19:**
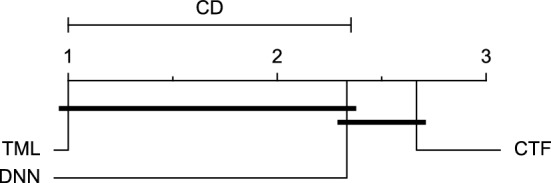
CD diagram of comparing different prediction models with multivariate input pairwisely over the data contributors of Ohio_2020 dataset for the 60-min prediction horizon based on MAE metric.

**Figure 20:**
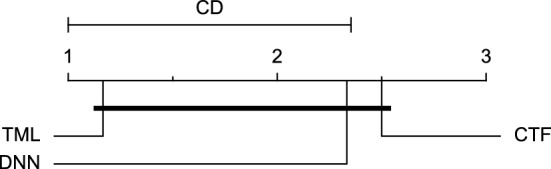
CD diagram of comparing different prediction models with multivariate input pairwisely over the data contributors of Ohio_2020 dataset for the 60-min prediction horizon based on MCC metric.

**Figure 21:**
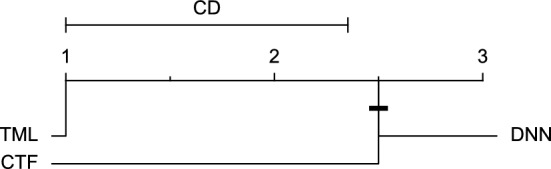
CD diagram of comparing different prediction models with multivariate input pairwisely over the data contributors of Ohio_2020 dataset for the 60-min prediction horizon based on SE metric.

Figures 11 and 12 show that the TML model, while performing similarly to the CTF model, outperformed the DNN model significantly for predicting BGL in the Ohio_2018 dataset 30 min in advance based on RMSE and MAE metrics, respectively. From Fig. 13, 14, and 21 it can be seen that the TML model statistically significantly outperformed both CTF and DNN models in the Ohio_2018 dataset based on SE metric for the 30-min prediction horizon and based on RMSE for the 60-min prediction horizon, and in Ohio_2020 dataset based on SE metric for the 60-min prediction horizon, respectively. Figures 15, 16, 17, 18, and 19 show that while the TML model performed similarly to the DNN model, it outperformed the CTF model significantly for the prediction horizon of 60 minutes in the Ohio_2018 dataset, based on MAE, MCC, and SE metrics, and also, in the Ohio_2020 dataset, based on RMSE and MAE metrics, respectively. Although based on Table 9, the result of the Friedman test calculated based on the MCC metric in the Ohio_2020 dataset for the 60-minute prediction horizon was significant, Fig. 20 shows that for the mentioned case, there was not a significant difference between BGL prediction performance using different prediction models. Also, Table 9 and Figs. 11, 12, 13, 14, 15, 16, 17, 18, 19, 20 and 21 reveal that the CTF and DNN models performed similarly for BGL prediction 30 and 60 min in advance using multivariate input in both cohorts.

#### Computational cost

When comparing different prediction models the computational cost of retraining them needs to be considered. The developed models do not have indefinite validity, and readjustments are required following changes in the BGL patterns. The computational costs of different prediction models on a standard laptop computer with a core i7 2.8 GHz processor, an NVIDIA GeForce GTX 1050 Ti GPU, and a 16 GB RAM were measured. Table 10 shows the average training time for different models of all data contributors in each cohort for each input and prediction horizon. The results illustrate that the TML model is the fastest and the DNN model is the slowest model for retraining purposes.

**Table 10:** The average training time (seconds) for models using different approaches across all patients in each cohort for each input and prediction horizon.

	Model	Univariate		Multivariate	
		PH:30 min	PH:60 min	PH:30 min	PH:60 min
Ohio_2018	CTF	277	289	502	530
	TML	10	11	20	16
	DNN	2057	2094	2051	2100
Ohio_2020	CTF	323	327	558	569
	TML	7	11	14	16
	DNN	1948	2099	1963	2149

*PH* prediction horizon, *CTF* classical time series forecasting, *TML* traditional machine learning, *DNN* deep neural network.

#### Summary

Review of the results presented in “Evaluation results”, “Statistical result”, and “Computational cost” shows that in more than half of the examined cases regarding evaluation metrics, prediction horizons, and datasets, especially using a univariate input, the three models performed comparably in BGL prediction. Among the rest of the cases, the TML model achieved the first rank with a significant superiority over at least one other model. In addition, the TML model was also the fastest model to be trained. The CTF and DNN models performed similarly for BGL prediction in all cases. Overall, the results suggest that the TML model is the superior approach for BGL prediction among the different examined data-driven models.

### Comparing models’ inputs

In this section, the effectiveness of univariate and multivariate inputs are compared using different CTF, TML, and DNN approaches, separately. The outcomes of statistical analyses are given and discussed in the following first section. Furthermore, a discussion about the ease and complexity of different inputs for collection and processing is presented. The results are then summarised to draw conclusions.

#### Statistical result

CTF approach Table 11 presents the Wilcoxon test p-values, based on each evaluation metric, prediction horizon, and cohort for examining whether the BGL prediction performance of the CTF model differs statistically significantly using different inputs. With a significance level of 5 %, the test outcomes show that exogenous variables did not affect the BGL prediction performance using the CTF model 60 min in advance in the Ohio_2018 dataset and both at 30 and 60 min in advance in the Ohio_2020 dataset based on all evaluation metrics. There is only one statistically significant difference (marked with bold font) between univariate and multivariate inputs using the CTF model, which is related to the RMSE metric for predicting the BGL 30 min in advance in the Ohio_2018 dataset.

**Table 11:** P-values of the Wilcoxon test for comparing univariate and multivariate input for the CTF model for BGL prediction 30 and 60 min in advance in Ohio_2018 and Ohio_2020 datasets.

	PH	RMSE	MAE	MCC	SE
Ohio_2018	30 min	**0.031**	0.062	0.438	0.094
	60 min	0.312	0.156	0.225	0.094
Ohio_2020	30 min	0.438	0.844	0.500	1.000
	60 min	0.219	0.562	0.686	0.219

*PH* prediction horizon, *RMSE* root mean square error, *MAE* mean absolute error, *MCC* Matthews correlation coefficient, *SE* surveillance error.

Considering Tables 4, 5, and 11, it can be concluded that, based on the RMSE metric, the CTF model performed worse with exogenous variables compared to univariate BGL prediction 30 min in advance over patients in Ohio_2018 dataset.

TML approach Table 12 displays p-values of the Wilcoxon test for examining if univariate or multivariate inputs can make a statistically significant difference in BGL prediction performance by applying the TML model. The test was performed over the data contributors of each cohort and was based on each evaluation metric and for each prediction horizon separately. With a significance level of five percent, the test outcome showed that the TML model predicted BGL significantly differently using different inputs for patients in Ohio_2018 dataset based on the SE metric for both prediction horizons. While the TML model performed similarly using different inputs in Ohio_2020 dataset for both prediction horizons.

**Table 12:** P-values of the Wilcoxon test for comparing univariate and multivariate input for the TML model for BGL prediction 30 and 60 min in advance in Ohio_2018 and Ohio_2020 datasets.

	PH	RMSE	MAE	MCC	SE
Ohio_2018	30 min	0.062	0.062	0.156	**0.031**
	60 min	0.062	0.062	0.156	**0.031**
Ohio_2020	30 min	0.438	0.562	0.312	0.844
	60 min	0.062	0.156	0.156	0.094

*PH* prediction horizon, *RMSE* root mean square error, *MAE* mean absolute error, *MCC* Matthews correlation coefficient, *SE* surveillance error.

Considering Tables 4, 5, and 12, it can be concluded that the TML model predicted BGL better according to SE metric using multivariate input compared to univariate input in Ohio_2018 dataset for both 30-minute and 60-minute prediction horizons.

DNN approach Table 13 displays the p-values obtained from the Wilcoxon test, which was performed based on each evaluation metric and for each prediction horizon, over the data contributors of each cohort. The test was conducted to determine whether univariate or multivariate input could make a significant difference in BGL prediction performance by applying the DNN model. The results showed that with a significance level of five percent, there was no statistically meaningful difference in the DNN model performance in predicting BGL using univariate or multivariate input in both datasets and for both prediction horizons, according to all examined evaluation metrics.

**Table 13:** P-values of the Wilcoxon test for comparing univariate and multivariate input of the DNN model for BGL prediction 30 and 60 min in advance in Ohio_2018 and Ohio_2020 datasets.

	PH	RMSE	MAE	MCC	SE
Ohio_2018	30 min	0.094	0.094	0.688	0.844
	60 min	0.312	0.562	0.094	0.562
Ohio_2020	30 min	0.844	0.844	0.688	0.688
	60 min	1.000	0.562	1.000	0.688

*PH* prediction horizon, *RMSE* root mean square error, *MAE* mean absolute error, *MCC* Matthews correlation coefficient, *SE* surveillance error.

#### Ease of data

Another important factor to be considered for comparing input for the BGL prediction task would be ease of data access. It is essential to consider how convenient data collection and preprocessing would be for each input. Developing a BGL prediction model using only data from a CGM sensor, which is a readily accessible tool for T1DM patients, requires automatic data collection with minimum human intervention and facilitates practicality of implementation regarding computational complications. In BGL prediction using a univariate input, there would be no need for extra effort and cost to acquire data from several sensors and modalities^15,16,18–20^. Also, multivariate input needs further data preprocessing steps, including data scaling up/down and data alignment. Moreover, according to Table 10, BGL prediction using multivariate input, needs more computational cost. Overall, univariate input is superior to multivariate input in terms of ease of data collection and processing.

#### Summary

According to the results in “Evaluation results”, “Statistical result”, and “Ease of data” the followings can be concluded. There was no conclusive evidence as to whether the use of univariate or multivariate input achieves better BGL prediction performance. With the CTF model, adding exogenous variables could make BGL predictions worse. In contrast, with the TML model, multivariate input may improve BGL prediction, or it may not significantly affect the performance of the DNN model. Also, BGL prediction performance was not significantly impacted by univariate or multivariate input in the Ohio_2020 cohort for the three forecasting models and both prediction horizons. Overall, the results reveal that considering exogenous variables, including Carb, Bolus, and activity, despite forcing more effort and cost, does not conclusively make a significant improvement in the performance of BGL prediction. It is important to note that this conclusion is based on the examined naive approaches of including exogenous variables. However, applying advanced data fusion approaches may alter the performance of the models and this conclusion.

## Conclusion

This work has comprehensively investigated the performance of different data-driven time series forecasting approaches including CTF, TML, and DNN, as well as the performance of different inputs, including univariate (BGL data only) and multivariate (BGL data along with Carb, Bolus, and activity) to provide insightful findings in the context of BGL prediction. The performance of different prediction approaches and inputs were compared for BGL prediction 30 and 60 min in advance. These investigations were performed using two Ohio_2018 and Ohio_2020 cohorts separately. Three prediction models related to the three different time series forecasting approaches were developed. The models were trained with a univariate input, and their counterparts were developed to cope with multivariate input. The different cases were evaluated using regression-based and clinical-based metrics followed by rigorous statistical analyses.

The obtained results showed that all three prediction models performed comparably in most cases. In the remaining cases, the TML model, which was also the fastest model to train, performed significantly better than the CTF, the DNN or both especially when using multivariate input. Moreover, comparing different inputs for each prediction model showed that adding extra variables, including Carb, Bolus, and activity and converting the univariate forecasting task to multivariate does not necessarily improve the BGL prediction significantly. In fact, different time series forecasting approaches perform differently for predicting BGL when dealing with multivariate data. The CTF model may perform worse by adding exogenous variables, the TML model may perform better using multivariate input, and the DNN model performs similarly using univariate or multivariate input. From the obtained results it is also can be inferred that to deploy the data of exogenous variables more effectively, information extraction and data fusion approaches may be required. Hence, investigating optimal approaches for fusion of extra variables with BGL is suggested as future work.

It is worth mentioning that in the current work, we investigated naive multivariate input for incorporating exogenous variables. Therefore, investigating effective approaches for leveraging affecting variables could be important to make a conclusive decision regarding the input of BGL prediction models. Hence, developing some approaches for effectively incorporating exogenous variables would be a future direction. Also, this work focused on data-driven approaches and using Physiological models for Carb and Bolus and developing hybrid prediction models are suggested. Moreover, it is worth noting that other potentially superior models for BGL prediction can be used in each forecasting group. Specifically, in the DNN approach, instead of LSTM, examining more advanced models with superior performance in handling complex temporal patterns (e.g. PatchMixer and SegRNN) could be suggested.

## Coding

Implementation of the methodologies was performed using Python 3.6, TensorFlow 1.15.0^55^, and Keras 2.2.5^56^, deploying the following packages: Pandas^57^, NumPy^58^, SciPy^59^, Sklearn^60^, statsmodels^61^, scikit-posthocs^62^, and cd-diagram^63^.

## Data Availability

The publicly available Ohio datasets^30,31^ used in this research are accessible, followed by requesting a data use agreement.
